# Pathophysiology of Blood–Brain Barrier Permeability Throughout the Different Stages of Ischemic Stroke and Its Implication on Hemorrhagic Transformation and Recovery

**DOI:** 10.3389/fneur.2020.594672

**Published:** 2020-12-09

**Authors:** Sara Bernardo-Castro, João André Sousa, Ana Brás, Carla Cecília, Bruno Rodrigues, Luciano Almendra, Cristina Machado, Gustavo Santo, Fernando Silva, Lino Ferreira, Isabel Santana, João Sargento-Freitas

**Affiliations:** ^1^Stroke Unit, Centro Hospitalar e Universitário de Coimbra, Coimbra, Portugal; ^2^Faculdade de Medicina da Universidade de Coimbra, Coimbra, Portugal

**Keywords:** blood-brain barrier, permeability, stroke, hemorrhagic transformation, pathophysiology

## Abstract

The blood–brain barrier (BBB) is a dynamic interface responsible for maintaining the central nervous system homeostasis. Its unique characteristics allow protecting the brain from unwanted compounds, but its impairment is involved in a vast number of pathological conditions. Disruption of the BBB and increase in its permeability are key in the development of several neurological diseases and have been extensively studied in stroke. Ischemic stroke is the most prevalent type of stroke and is characterized by a myriad of pathological events triggered by an arterial occlusion that can eventually lead to fatal outcomes such as hemorrhagic transformation (HT). BBB permeability seems to follow a multiphasic pattern throughout the different stroke stages that have been associated with distinct biological substrates. In the hyperacute stage, sudden hypoxia damages the BBB, leading to cytotoxic edema and increased permeability; in the acute stage, the neuroinflammatory response aggravates the BBB injury, leading to higher permeability and a consequent risk of HT that can be motivated by reperfusion therapy; in the subacute stage (1–3 weeks), repair mechanisms take place, especially neoangiogenesis. Immature vessels show leaky BBB, but this permeability has been associated with improved clinical recovery. In the chronic stage (>6 weeks), an increase of BBB restoration factors leads the barrier to start decreasing its permeability. Nonetheless, permeability will persist to some degree several weeks after injury. Understanding the mechanisms behind BBB dysregulation and HT pathophysiology could potentially help guide acute stroke care decisions and the development of new therapeutic targets; however, effective translation into clinical practice is still lacking. In this review, we will address the different pathological and physiological repair mechanisms involved in BBB permeability through the different stages of ischemic stroke and their role in the development of HT and stroke recovery.

## Introduction

The blood–brain barrier (BBB) is a dynamic physiological structure that constitutes an interface between the vasculature system and the neural tissues, regulating diverse processes such as cerebral blood flow and angiogenesis, neuronal development, and synaptic activity (1). It also acts as a physical and metabolic (2) barrier that regulates the transport of substances in a bi-directional way (3) and protects the central nervous system (CNS) from unwanted compounds playing a crucial role in maintaining its homeostasis (2, 3). The precise knowledge of the structure and functioning mechanisms of the BBB on physiological conditions is key to understand how it reacts to different situations and pathologies (4). One rather complex condition is acute ischemic stroke (AIS). This pathology is characterized by different hemodynamic stages where BBB permeability (BBBP) can either be a friend or a foe, favoring hemorrhagic transformation (HT) on the one hand and enhancing neoangiogenesis and allowing the delivery of potentially therapeutic agents whose access to the CNS would be otherwise impossible on the other hand. In AIS, we can define hyperacute (<6 h), acute (6–72 h), subacute (>72 h), and chronic stages (>6 weeks). Each phase has its own particular BBB status, with distinct pathological backgrounds and often contradictory clinical consequences. Ultimately, BBB disruption plays a key modulator and precipitant role in HT, recognized as the most devastating complication after an ischemic stroke.

### Structure of the Blood–Brain Barrier: Neurovascular Unit and the Junctional Complex

**Figure 1:**
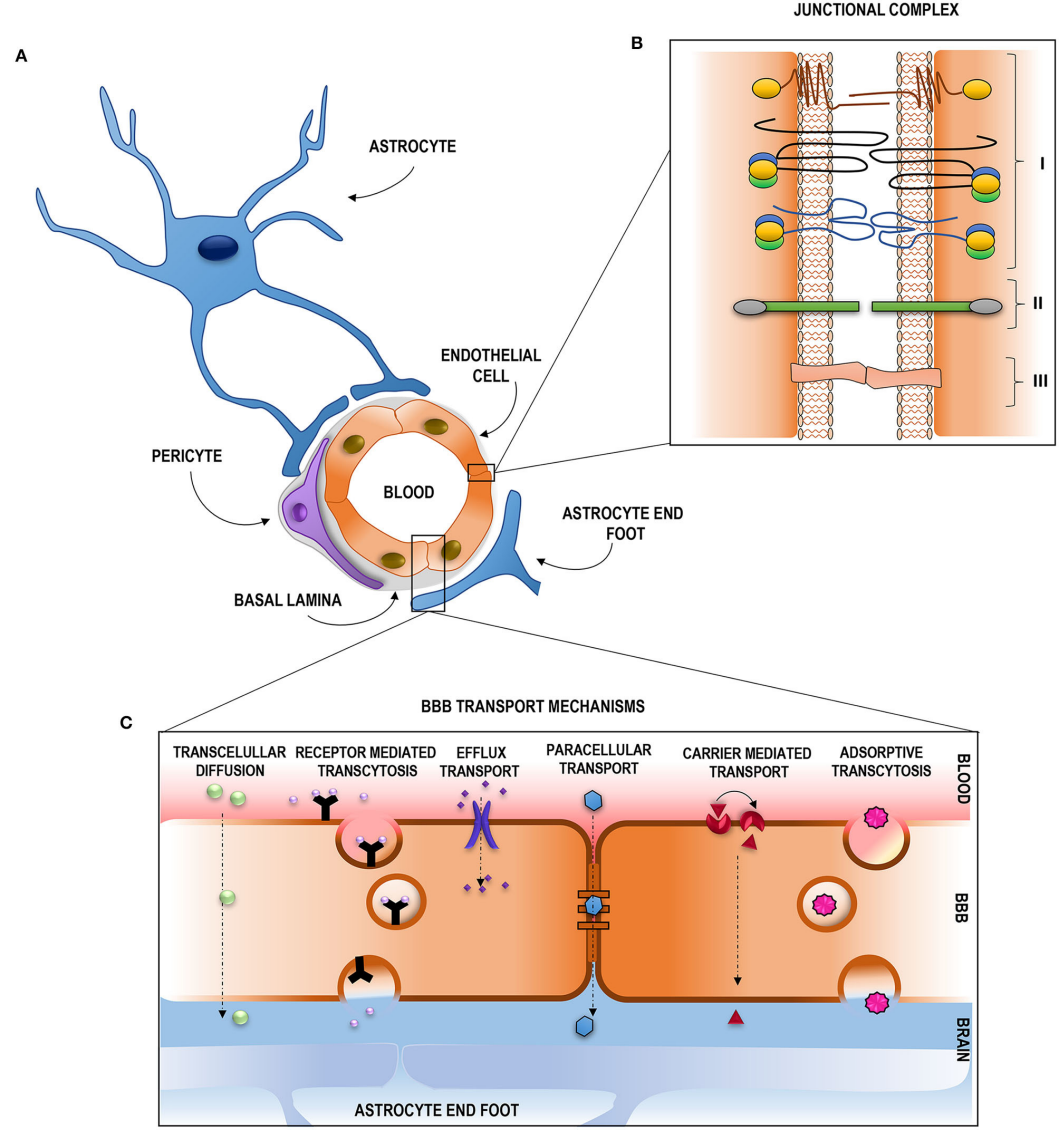
Structural composition and transport pathways across the blood–brain barrier. **(A)** Representation of the distribution of the NVU components. **(B)** Junctional complex of the brain endotelial cells: (I) Tight junctions: composed by JAMS, caludins and occludins. ZO proteins are represented in yellow (ZO-1), green (ZO-2) and blue (ZO-3); (II) Adherens Junction, cadhenins represented in green and caterins in gray; (III) GAP junctions. **(C)** Schematic representation of the transport pathways across the BBB.

The selective and protective features of the BBB are due to its special structural composition; Figure 1 shows a schematic representation. The main physical barrier between the blood and the CNS is composed of the BBB endothelial cells (BECs) of the blood vessels (5), but to fulfill all BBB characteristics, they function along with other components. BECs are surrounded by pericytes and astrocytes (through their foot processes), and the basement membrane is composed of extracellular matrix components (EMCs) (1, 4, 6–8), constituting a continuous stratum that separates the vasculature and the neural tissue (4). The relationship between all these components constitutes a dynamic functional unit called the neurovascular unit (NVU) (4, 6, 7).

#### BBB Endothelial Cells

BECs constitute the innermost luminal component of the BBB (4). Their unique properties make them distinguishable from other peripheral endothelial cells (4) and allow them to strictly regulate ion movement across CNS (9). These cells contain a higher number of mitochondria, allowing the generation of greater amounts of biological energy required to maintain BBB integrity (10) and augment its selective molecular permeability (4) BECs are polarized (8) and display numerous receptors, ion channels, and surface transport proteins along with limited vesicular transport (1, 4, 11). They also possess extremely low levels of leucocyte adhesion molecules which hamper the infiltration of immune cells into the CNS and a negative surface charge that repels negatively charged compounds (1). All these features strictly regulate solute permeability into the CNS, along with the presence of the junctional complex, which binds BECs to each other (12).

#### Junctional Complex

The junctional complex is compromised by tight junctions (TJs), adherens junctions (AJs), and GAP junctions (GJs) (12, 13). A schematic representation is given in Figure 1. TJs reduce the permeation of polar solutes into the brain extracellular fluids and limit the passage of proteins and lipids located at the apico-lateral membrane (13–15). They are composed of a series of transmembrane adhesion proteins, cytoplasmatic/scaffolding proteins, and an actin cytoskeleton. Transmembrane proteins compromise claudins, which are the primary sealing proteins of TJs, occludins, acting as regulators and as a platform for signaling processes, and junctional adhesion molecules, key proteins on tubule formation and in leukocyte adhesion and transmigration. The scaffolding proteins provide a link between the transmembrane proteins and the actin cytoskeleton and participate in intracellular signaling. This group of proteins is formed by the zonula occludes proteins, and they are essential for claudin strands, occludins, and JAM assembly and for anchoring these proteins to the actin cytoskeleton which delivers essential physical support for the complex. AJs hold the cells together, giving the tissue structural support (14), and are mainly composed of cadherins, transmembrane proteins responsible for the adhesion between cells, and catenins, cytoplasmatic proteins that support cadherin association and regulate out–in processes. GJs are crucial for intercellular communication and are composed of members of the connexin family (Cx) (12). Disruption of this junctional complex assembly or function directly affects the BBB characteristics, mainly its permeability (12).

#### Basal Membrane

Surrounding BECs, we can find the basement membrane (BM). The BM is the acellular component of the NVU (1) compromised by a specialized layer of EMCs, including type IV collagen, laminin, nidogen, and heparin sulfate proteoglycans (9, 10). The BM provides an anchor for the rest of the cellular components, thus mediating in their crosstalk and providing microvascular stability (1, 9). The disruption of BM will lead to damaged TJs and compromised BBB integrity (1).

#### Pericytes

Embedded within the BM are pericytes (1, 9). Pericytes are critical for maintaining BBB integrity (8). They form peg–socket-type junctions with BECs (1, 9, 16) which prevent leucocyte infiltration through the BM into the CNS (1). Pericytes synthesize some important EMCs for BM formation (8) and are able to modulate capillary diameter through the expression of contractile proteins (1, 17). In addition, pericytes can play a similar role to macrophages (18), implying a phagocytic function able to degrade cell debris and erythrocytes following leakage after BBB disruption (1, 18).

#### Astrocytes

With their end-foots completely covering cerebral blood vessels (1, 9, 19) are astrocytes. Astrocytes are the major glial cells of the NVU (1, 9) proving a link between the neural system and vasculature (20) and assuming a central role in dynamic CNS signaling (1). Astrocyte end-foots express a high-density of orthogonal arrays of particles, among which we can find aquaporin IV, critical for regulating water homeostasis in the CNS (9, 21). This neuro-vascular coupling enables astrocytes to adjust CBF in response to local neurons by eliciting vasoconstriction and vasodilation of brain vessels (22) regulating the contraction and dilation of pericytes surrounding capillaries. Astrocytes also contribute to neuronal functions such as synaptic plasticity (23) and provision of energy substrate (19). Thus, the high relationship between astrocytes and vasculature is essential in maintaining BBB integrity (19, 24).

The NVU is a dynamic structure in close contact with, among others, immune cells. In this context, it is of special relevance to mention microglia cells.

#### Microglia

Microglia are tissue-resident macrophages (25) and are the most abundant immune cells in the CNS (26). They participate in the NVU by occasionally contacting with microvessels (24) and are the primary mediators of CNS inflammatory response (26). Microglia cells detect the very first signs of tissue damage (27) and are able to vary their phenotype in the presence of any threat. Along this, microglia have a pivotal role in the maintenance of CNS homeostasis (27), constantly surveying and screening the microenvironment within the NVU (28). Furthermore, microglia communicates in an active and dynamic way with neurons (28, 29), playing a crucial role in supporting their functions (28).

Recognition of the NVU is of high importance to reach a better understanding of brain physiology and its behavior in different pathologies such as ischemic brain injury (6). The close contact between the NVU, neurons, microglia, and other immune cells (10), along with the functional interactions and signaling between all the components (30), confers the BBB its unique characteristics, and in order to maintain its correct functioning, the neural environment must be preserved. This requires the precise regulation of molecule and ion transport between the blood and the brain (4).

### Transport Across the Blood–Brain Barrier

Despite this efficient barrier function for preventing the entrance of non-desirable compounds, the ECs of a healthy BBB also act as a filter, allowing a selective exchange of solutes and regulatory factors between the blood and the brain through a number of highly controlled routes (30–32). These routes can be divided into two main pathways: paracellular and transcellular (33, 34) (Figure 1). The paracellular transport occurs by passive diffusion through the TJs, while the transcellular transport pathway occurs *via* the BECs *per se* (32) and can either be energy dependent (active) or not energy dependent (passive) (34). The passive transcellular pathway includes transcellular diffusion, while the active transcellular pathway includes receptor-mediated transcytosis (RMT), active efflux transport, and adsorptive-mediated transcytosis (32, 35). Carrier-mediated transport (CMT) can be either energy dependent or energy independent (34).

#### Paracellular Pathway

The paracellular pathway occurs between cells by the passive diffusion of low molecular mass hydrophilic molecules (32, 33, 36, 37) depending on the electrochemical, hydrostatic, and osmotic gradient (34, 38). Nonetheless, in the CNS, this transport is highly restricted and conditioned by the TJs (30, 38) and is, therefore, negligible (32). Thus, the vast majority of molecules have to use the transcellular pathway to cross the BBB.

#### Transcellular Pathway

##### Transcellular Diffusion

Transcellular diffusion takes place at the luminal and abluminal membrane of BECs (38) and is restricted to gases such O_2_ and CO_2_ (2) and small lipophilic molecules (36, 37) of <400 Da (8) and <8 hydrogen bonds (39).

##### Carrier-Mediated Transport

CMT can either be active or passive (34), and it allows the exchange of molecules between blood and CNS through the BBB *via* substrate-specific transporters (8, 34). GLUT-1, large neutral amino acid transporters, and nucleoside transporters are among the CMT proteins of great importance in BBB homeostasis maintenance (39).

##### Receptor-Mediated Transcytosis

RMT is the main pathway used for the uptake of molecules that do not possess a specific carrier (39), such as hormones and high molecular mass proteins (32, 34). These molecules bind to the specific receptor on the cell surface, resulting in the formation of endocytic vesicles that will cross the BBB to release the ligand, allowing the receptor to be recycled (30). The transferrin receptor and the low-density lipoprotein are good examples of this pathway (32).

##### Efflux Transport

Efflux pumps are responsible for the removal of substances out of the CNS into the systemic circulation in order to prevent the accumulation of compounds that have gone through the BBB (34, 38). Among these efflux pumps, we can find proteins belonging to the ATP-dependent binding cassette (ABC) transporter superfamily such as P-glycoprotein (P-gp or ABCB1) (37, 39).

##### Adsorptive Transcytosis

Adsorptive transcytosis relies on the non-specific transport of positively charged substrates, such as cationized albumin, when they react with the negatively charged surface of BECs (32, 38).

After briefly reviewing the physiological functions of BBB and NVU, it is clear that the strict regulation of the BBB plays a fundamental contribution to the maintenance of brain homeostasis, triggering a vast number of pathological consequences when any of its components is altered (6). Such alterations can lead to a disruption of the BBB and an increased BBBP which is linked to the pathophysiology of many neurological disorders including stroke (35).

### Blood–Brain Barrier and Stroke

A total of 14 million people suffer from stroke worldwide; 5.5 million of them die and another 5 million stay permanently disabled (40, 41), placing this disease as the second leading cause of mortality and morbidity worldwide (40–43). Then, 86% of all strokes are of ischemic nature (11), and they occur as a consequence of the interruption or severe reduction of blood flow and oxygen in cerebral arteries (44). This initial occlusion causes a myriad of dynamically interconnected pathophysiological events (35) that start with the onset of the ischemic insult (45) and follow a time-dependent progression through the different stroke stages. These events normally overlap (46) and can eventually lead to the destruction and/or dysfunction of brain cells, causing neurological deficits (47). Nowadays, the only approved and effective treatment to try to avoid this situation is recanalization therapy to restore the normal blood flow, but the narrow therapeutic window of the disease limits its use to ~5% of patients. Treating patients outside this window could contribute to additional tissue damage and an increase in the risk of HT (48, 49).

**Figure 2:**
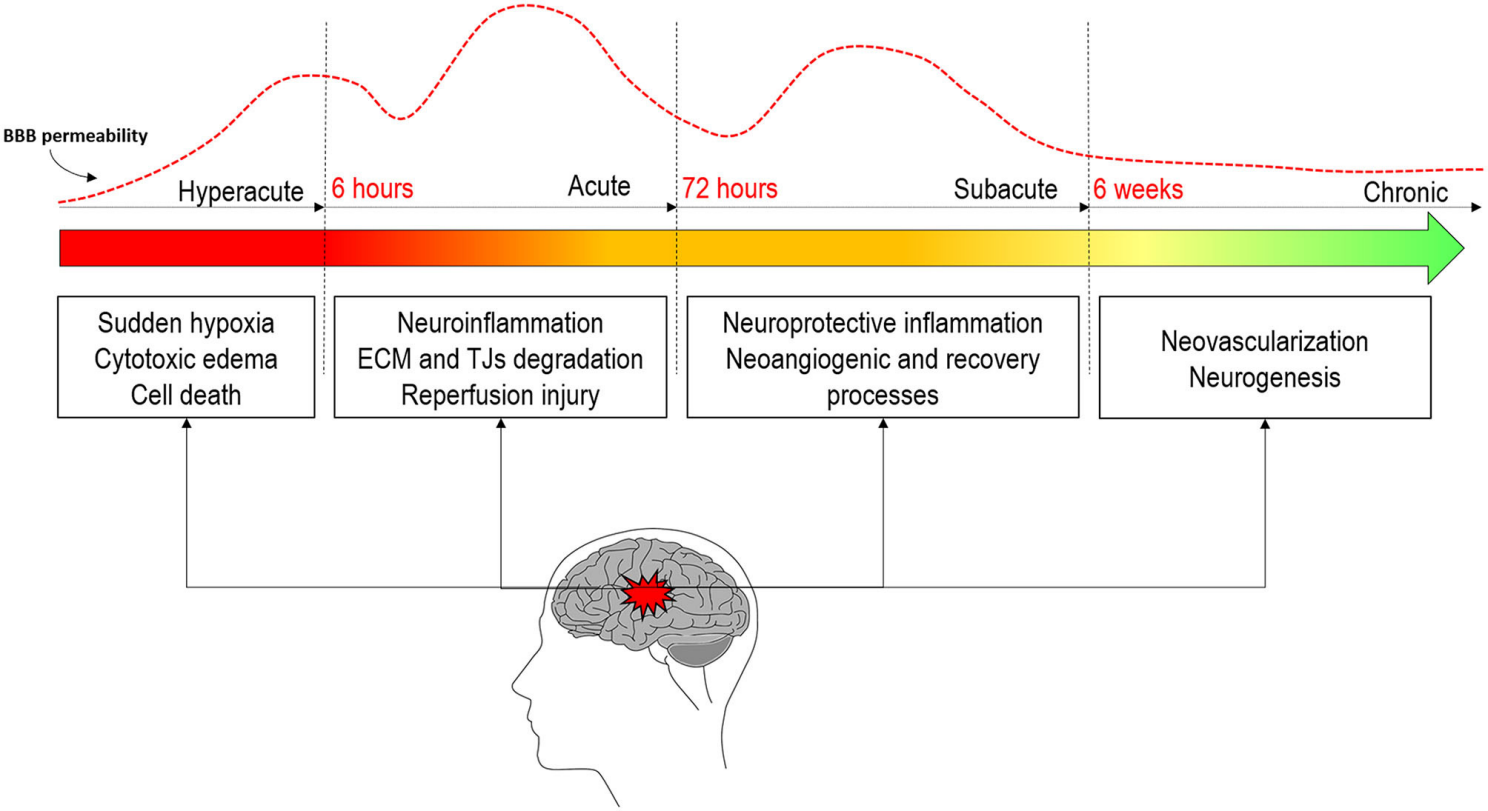
Evolution of blood–brain barrier permeability throughout the different stages of stroke and the main pathophysiological processes in each stage.

One of the major events taking place in this pathophysiological response is the disruption of the BBB (3). It appears soon after the onset of artery occlusion and continues for several days to weeks after stroke (6). BBB disruption can be associated with reperfusion and is consequently attributed to dysfunctional TJs and endothelial damage, leading to increased permeability of the affected vessels (50). But rather than being solely a consequence of injury, BBB disruption also contributes to it (51) and is usually associated with poor clinical prognosis (3, 42, 49). The increase in BBBP enables the passage of molecules, fluids, and blood into the brain (52) and follows a complex time-course progression mediated by complex pathophysiological processes (53) that go from initial to secondary injury and to tissue repair and later regenerative events (54). The concrete pathways underlining the BBB dysfunction and repair in stroke are yet unclear. Recent studies are accumulating evidence proposing that the fine-tuning of these complex pathways is regulated by the action of microRNAs (miRNAs). miRNAs are endogenous, single-stranded, non-coding RNAs which inhibit protein synthesis by either mRNA degradation or transient translational arrest, allowing them to regulate most biological processes from apoptosis, inflammation, or oxidative stress to angiogenesis and neurogenesis. In fact, it has been shown that miRNAs are key in the BBBP regulation (55, 56). Figure 2 shows a schematic representation of the main processes driving BBBP.

Traditionally, the dynamics of the BBB were thought to occur in a biphasic “open–close–open” process (57, 58), but the inconsistency in the opening/closing times has lead recent literature to propose a more continuous opening with biphasic peaks but without the BBB closing in between (46, 59–62). The first opening has been documented to occur in the hyperacute stage of stroke within the first 6 h after onset in both animal (46, 60–62) and human studies (63–65). This initial permeability is thought to be due to the sudden hypoxia of different brain cells and early BBB disruption (11). During the next 72/96 h, in the acute phase of stroke, the neuroinflammation processes motivated by the first cytotoxic events will further rupture the BBB (54). It is at this stage where the second peak of permeability is usually observed (46, 60, 62). This greater increase in permeability will lead to a higher risk of HT motivated by several mechanisms, among which is reperfusion (47, 66). Further longitudinal studies in animal models of stroke have demonstrated that permeability remains increased up to weeks after stroke (61, 62, 67). Some human studies have also demonstrated elevated permeability after 1 week (63, 68, 69). This suggests that BBB stays opened during the subacute and the chronic stages of stroke. This late opening is believed to be correlated with regenerative processes that will improve recovery and wound healing rather than contribute to pathology.

This review aims to give a meticulous view on the complexity of the cellular and molecular mechanisms that lead to the increase of BBBP during the different phases of stroke and its clinical implication on the development of reperfusion injury and HT, as well as the recovery processes derived from it. This knowledge will hopefully offer guidance on future novel treatments that directly target BBB permeability either by preserving its integrity or as a vehicle for drug delivery as well as a better understanding of how the BBB status may influence acute stroke care decisions.

## The Blood–Brain Barrier in the Hyperacute Phase of Stroke

As cerebral blood flow (CBF) is impaired, delivery of oxygen and glucose—two essential substances to brain metabolism—is compromised. Adenosine triphosphate (ATP) levels reduce in the ischemic brain tissue, and ionic transporters Na^+^-K^+^-ATPase and Ca^2+^-ATPase lack the substrate for its normal functioning. Na^+^ accumulates within the cell, driving the movement of fluid inwards and resulting in oncotic cell swelling termed cytotoxic edema (70). This occurs just as CBF is below 30/100 mg^*^min and can be detected immediately after an arterial occlusion through the decrease of the apparent diffusion coefficient of water which, in turn, is responsible for the increase of signal intensity in diffusion-weighted magnetic resonance imaging (71–73). At this phase, the BBB is mainly intact.

Uptake of Na^+^ in endothelial ion transporters through Na–K–Cl cotransporter and Na/H exchanger that does not depend on ATP is not effectively counterbalanced by Na^+^ secretion, leading to endothelial cell swelling and BBB breakdown (11). An increase in intracellular Ca^+^ is promoted by a failing Ca^2+^-ATPase coupled with a functioning Na^+^/Ca^2+^exchanger with plenty of intracellular Na^+^. This intracellular Ca^+^ disturbs cellular mechanisms and accelerates cell death through the toxicity of high concentrations of glutamate and dopamine and the activation of several Ca^2+^-dependent catalytic enzymes (74). Glutamate excitotoxicity coupled with cellular depolarization overstimulates metabotropic and ionotropic glutamate receptors, AMPA and NMDA receptors, disrupting calcium homeostasis even more. Ca^2+^-induced mitochondria dysfunction leads to reactive oxygen species (ROS) generation such as superoxide anions (75); but not only ions are responsible for this early BBB disruption, other mediators such as aquaporins, matrix metalloproteases (MMP), inflammatory cells, bradykinin, vascular endothelial growth factor, and nitric oxide synthase appear to have a role (7, 76, 77). In fact, MMPs seem to be a key player in BBB disruption as they directly degrade TJ proteins and ECM components (53). As the BBB breakdown occurs, within minutes to hours of ischemic onset (78), macromolecules such as proteins exit the vasculature and enter the brain extracellular space, exerting an osmotic gradient, pulling water, and generating vasogenic edema. A phase of ionic edema is also sometimes referred to as an intermediate between cytotoxic and vasogenic edema, corresponding to the formation of extracellular edema with an undamaged BBB and impermeable BBB to larger molecules such as proteins (70, 71). Since white matter is more compliant than gray matter, vasogenic edema tends to affect the former more. This occurs in the first 4–6 h after the initial vascular insult and can be identified by conventional MRI T2-weighted images and fluid-attenuated inversion recovery sequences (79, 80). A larger volume of water is mobilized in vasogenic edema compared to cytotoxic edema, paving the way for brain swelling, increased intracranial pressure, herniation, and additional ischemic injuries due to an imbalance between brain pressure and capillary pressure. Brain edema is a life-threatening complication and a leading cause of early death after stroke (81). Such phenomenon is commonly associated with infarctions involving the whole middle cerebral artery (MCA) territory, a condition that accounts for 10% of all ischemic strokes (82). In those malignant MCA infarctions, edema generally peaks between 48 and 96 h after the onset, with a fatal outcome in 80% of patients in the first week (76, 82). Several anti-edematous and intracranial pressure-lowering therapies have been proposed, including osmotherapy, steroids, hyperventilation, and early decompressive craniectomy, the latter being effective in mortality reduction (83). The overall very poor prognosis has recently led investigators to evaluate, in randomized clinical trials, the potential benefit of endovascular recanalization in patients with acute ischemic stroke due to a large vessel occlusion in the distal internal carotid artery and MCA M1 segment who have already a large core of infarction [Alberta Stroke Program Early CT Score (ASPECTS) < 6]. Patients with low ASPECTS may still benefit from endovascular recanalization since it may reduce the risk of edema formation and malignant MCA infarction (84). SELECT-2 (NCT03876457), IN EXTREMIS-LASTE (85), TENSION (NCT03094715), and TESLA (NCT03805308) trials are currently underway to evaluate this hypothesis.

In this context, HT occurs as an end-stage endothelial dysfunction, with a compromise of capillary integrity and extravasation of blood into the brain parenchyma. It is the sum of ischemia plus reperfusion.

### Reperfusion

The reestablishment of blood flow in a previously occluded vascular bed is a double-edged sword. It may warrant the survival of the surrounding penumbra, but at the same time, it might contribute to the flow of water and osmotic solutes through the ruptured BBB. Reperfusion is a three-stage process that may occur spontaneously and can be stimulated or anticipated by recanalization therapies with reopening of the occluded artery (78, 86). In this process, there is a first stage of reactive hyperemia with loss of cerebral vasoregulation associated with cytotoxic edema. Then, there is a following stage of hypoperfusion in relation to a reactive microvasculature obstruction through endothelial and astrocyte end-feet swelling, microvilli formation, and inflammatory activation that further aggravates BBB breakdown (86). This corresponds to a no-reflow effect and a phase of ischemic stunning of the brain, a term coined in cardiology to describe myocardium contractile depression after reperfusion of coronary occlusion (87, 88). Interestingly, it has been shown that, after ischemic injury, pericytes contract and remain like that even after the complete reopening of the occluded artery, this being one of the responsible factors for the non-reflow effect after recanalization therapy (89) that negatively affects tissue survival (90). After this hypoperfusion, there is an increase of paracellular permeability mediated by MMP which predominantly occurs between 3 to 8 h (MMP-2) and 18 to 96 h (MMP-3 and MMP-9) after the initial reperfusion. This latter period is associated with vasogenic edema and angiogenesis (86).

As described, BBB disruption is intrinsically part of the ischemia-reperfusion continuum and a fundamental but not sufficient factor to the most severe clinical presentation of reperfusion injury which is intracerebral hemorrhage. One important aspect here is timing. Although reperfusion is absolutely necessary for tissue survival, it can contribute to additional tissue damage (47, 78), and the later spontaneous recanalization is achieved, the higher is the risk of HT (91). The same is true to thrombolysis, as initiating recombinant tissue plasminogen activator (rTPA) treatment beyond the recommended 4.5 h from symptom has been associated with adverse effects, particularly HT (92). This situation will be discussed in the later sections of this review.

The hyperacute management of ischemic stroke goes beyond recanalization therapy. Common concurrent conditions and/or complications need to be tackled as a way of avoiding additional harm to the brain and thus promoting neuroprotection.

### The Importance of Comorbidities in the Acute Management of Stroke

Glycemia, oxygen, blood pressure, and temperature need to be within certain thresholds. Hyperglycemia and hypoglycemia are both contributors to BBB dysfunction in infarct regions after reperfusion, mediated by elevations in the expression of MMP-2/-9 and decrease of TJ proteins including occludin, claudin-5, and ZO-1 (93). At this early stage of stroke, hyperglycemia also causes BBB disruption, again mediated by MMP-2/9 extracellular degradation, caveolin-1-mediated intracellular translocation, and autophagy-lysosome-mediated degradation of ZO-1 protein (94). Hypoxia is another insult that alters BBB integrity, increasing its permeability through MMP-9-dependent loss of tight junctions with disrupted continuity of occludin and ZO-1 (95) and through the generation of reactive oxygen species (96). Hyperthermia is another significant and independent contributor to BBBP (95, 97).

## Blood–Brain Barrier Permeability in the Acute Phase of Stroke and Its Implication on Hemorrhagic Transformation

After the first six hyperacute hours and during the next 72/96 h, the acute stage of stroke will take place. This stage is critical for saving the surrounding area of the ischemic core, known as peri-infarct tissue, as the cell death processes initiated in the hyperacute phase can expand and become a part of the ischemic core if not salvaged by the reperfusion therapy (54). The progression of brain ischemia over time from the infarct core to the penumbra and peri-infarct tissue involves secondary injury cascades. Delayed cell injury in the penumbra occurs with inflammation and free radical generation which will not only produce secondary damage and modify the extracellular matrix but also generate the signals for neural repair in later stages (54).

Over the course of days to weeks, the neuroinflammatory response takes place (98), becoming the main factor for increased BBBP (7). Recent evidence points that inflammatory processes in stroke are regulated by miRNAs. Neuroinflammation can be induced by miRNAs such as miR-155 or suppressed by miRNAs like miR-146a, miR-124, or miR-21. Other miRNAs can have the capacity of suppressing or promoting the inflammatory response, such as the case of the let-7 family (99). Ischemia-induced cell death, cell debris, and increased ROS produced in the hyperacute stage lead to neuroinflammation by activating resident microglia and astrocytes (100, 101). This activation has been shown to occur 4–6 h after occlusion in animal models of stroke (102). At 24 h, the microglial reaction is well developed (102), and it reaches its proliferation peak at 48–72 h after focal cerebral ischemia, lasting for several weeks (98). Of important note is that, upon activation, microglia can acquire two phenotypes depending on their polarization (103): M1 phenotype, the classically activated microglia that contributes to neuroinflammation and increased BBBP, and M2 phenotype, which will have an important role in recovery (6, 100, 101, 104). Pro-inflammatory microglia is capable of releasing cytotoxic compounds (101) such as nitric oxide and inflammatory cytokines like IL-1β, IL-1α, TNF-α, and IL-6, favoring BBB disruption and the increase of its permeability (27, 98, 100, 103). These pro-inflammatory cytokines play crucial roles in the neuroinflammatory cascade that will affect the disruption and permeability of the BBB. The activation of TNF-α receptors has a neurotoxic repercussion that leads, among other things, to the activation of apoptotic factors and MMPs (105). Moreover, TNF-α can disrupt the BBB by reducing claudin-5, occluding, and ZO-1 expression, affecting the stability of TJs (35). On its side, IL-1 induces endothelial activation, leading to an increase in cytokines/chemokines and MMP-9 production, which comes along with BBB disruption and immune cell infiltration (105).

The production and the upregulation of MMP-9 are extremely important in stroke development and outcome. This metalloproteinase plays a crucial role in BBB breakdown. MMP-9 belongs to the gelatinases group in the metalloproteins family (106). It has a pivotal role in the proteolytic degradation of the ECM components of the BBB and is capable of digesting TJ proteins such as occludin and claudin, contributing to BBB disruption and permeability increase (107) since the degradation of essential components such as laminin, fibronectin, collagens, or proteoglycans destabilizes structural support for the BBB, producing leakage and breakdown (108). Its expression is rapidly upregulated in ischemic injury (109), reaching high peaks of activity at around 24–48 h (109, 110). The high activity of MMP-9 within the acute phase of ischemic stroke has been reported to increase the risk of secondary bleeding, and its presence in AIS patient's serum is correlated with worse clinical outcomes. In fact, MMP-9 degradation of the matrix is a major contributor to intracranial hemorrhage (108).

On its side, IL-6 plasma levels also correlate with stroke severity and poor clinical outcome (105). It can be detected in the first hours after stroke onset but reaches its peak at 24 h, remaining detectable up to 14 days (111). IL-6 produces gliosis, activates endothelial cells, and increases BBB damage in stroke (7). Furthermore, it actively contributes to the synthesis and release of some chemokines (105).

Chemokines are cytokines that, acting on its inflammatory function (103), are able to attract infiltrating leukocytes from circulating blood (112), contributing to aggravate this situation (100, 101). They are released in response to the action of the inflammatory cytokines by damaged CNS cells (105). Three of the most studied chemokines in the human neuroinflammatory response are the macrophage inflammatory protein1-α (MIP-1α or CCL3), the monocyte chemotactic protein-1 (MCP-1 or CCL2), and CCL5 or RANTES (103, 113) While CCL2 and CCL3 are associated with an enlarged ischemic territory, monocyte accumulation, and microglial activation in the injured brain tissue, respectively, CCL5 has been shown to be a potent pro-inflammatory chemokine linked to a greater BBB disruption, possibly by enhancing MMP-9 activity (103).

Another factor that has been shown to be key to the progression of ischemic brain damage is cyclooxygenase (COX)-2 from the COX inflammatory enzymes. It contributes to BBB damage as part of a secondary inflammatory response from 24 to 72 h after the initial insult (114).

All of these pathological neuroinflammatory responses will, therefore, lead to the rupture of the BBB components, and TJ dysfunction is perpetuated; thus, paracellular permeability increases (35), allowing the penetration of, among other molecules, thousands of peripheral immune cells into the brain (6, 100). These peripheral immune cells have the capacity of augmenting neuroinflammation by producing cytotoxic compounds that will join the damaged CNS cells and worsen BBB disruption (6). Among the infiltrating immune cells, neutrophils are of special relevance. Neutrophils are the primary responders after ischemic injury (115). The infiltration of neutrophils into the brain is aided by adhesion molecules *via* their attachment to the endothelial wall, thereby stimulating and facilitating diapedesis through the vessel wall to the site of ischemic brain injury (101). Neutrophils reach their peak around 2–4 days post-injury and then decrease (101). The presence of neutrophils in peripheral blood has been associated with worse clinical outcomes in AIS patients (116, 117). This may be associated with the fact that neutrophils show an important role in basal lamina degradation and hence in BBB disruption, most likely because they produce MMP-9 (117–120).

As all these processes are taking place, the BBB becomes more permeable and thus more likely to completely rupture. This scenario could lead to the extravasation of high amounts of blood into the brain, causing one of the most common and worst outcomes of ischemic stroke, HT.

## Hemorrhagic Transformation: The Role of BBB Permeability

HT is a common and serious complication of AIS occurring in a total of 30–40% of clinical cases (81, 121). It can happen spontaneously as a natural evolution of stroke or precipitated by reperfusion therapy (121, 122). In any of the cases, HT occurs when cerebral blood flow is restored to damaged blood vessels weakened by ischemic stroke (47, 66). In fact, the disruption and leakiness of the BBB on pretreatment imaging is correlated with the severity of HT (123). Furthermore, a higher BBBP is associated with not only the severity of but also the likelihood to develop HT (64, 124, 125). This association has been shown by two systematic reviews studying the prediction of HT with MRI (126) and computed tomography (CT) imaging (127). The pathophysiology of HT is multifactorial and has not yet been clearly elucidated, but it is linked to processes that alter the integrity of the BBB and basal lamina matrix (121). Among the factors that are implicated in this process, we can find matrix metalloproteinases, inflammation, vascular endothelial growth factor, nitric oxide synthase, and free oxygen radicals (81).

**Figure 3:**
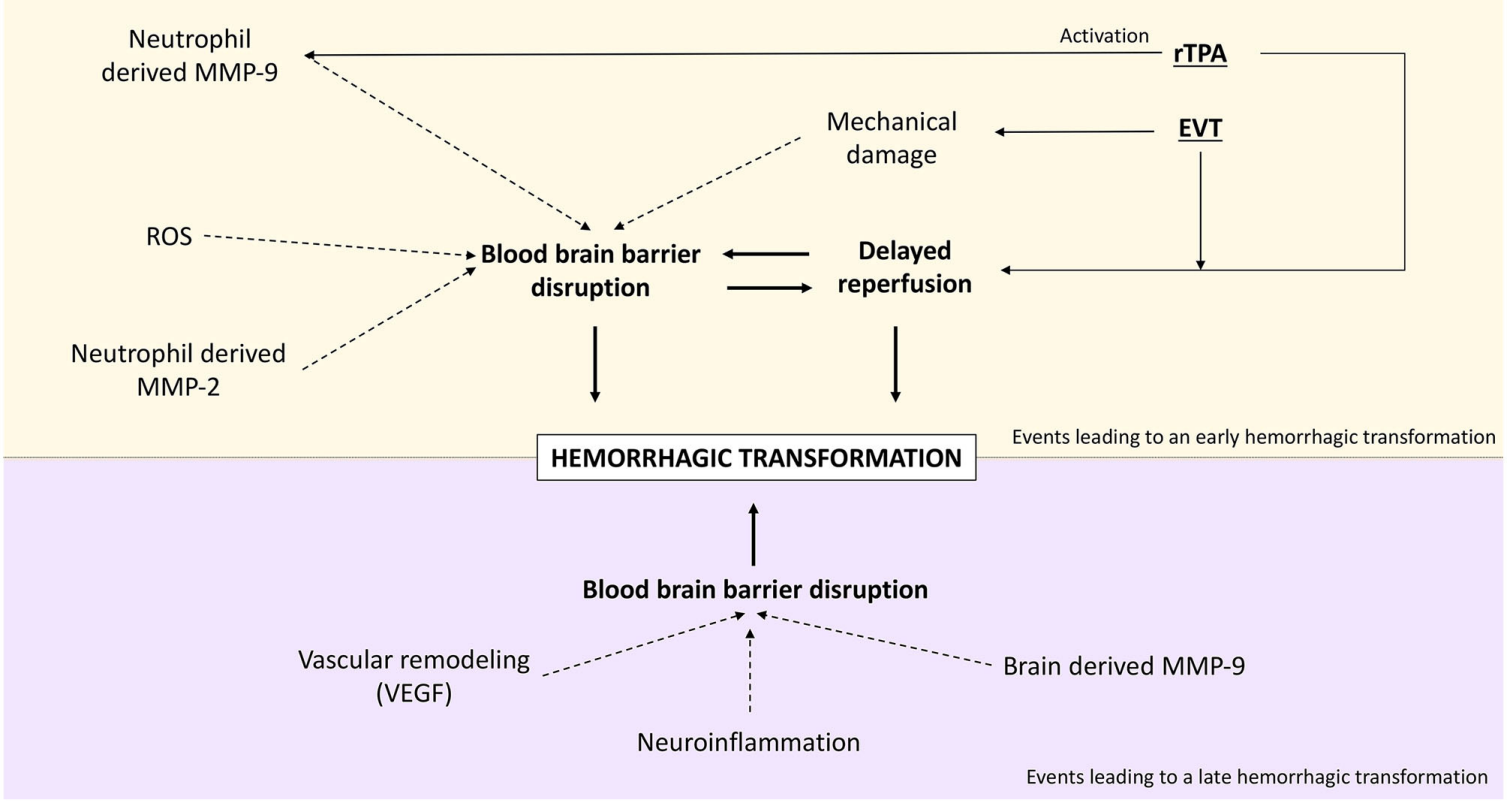
Main events leading to hemorrhagic transformation after stroke.

HT occurs in an undetermined period of time that varies from a few hours to even weeks after stroke. It has been hypothesized that the mechanisms that drive the early appearance of HT (<36 h) are different from those driving delayed HT (66). In short, early HT correlates with earlier BBB disruption which is often due to reperfusion treatment (128). In fact, HT appearing on the first 36 h after treatment is directly correlated to reperfusion (122). On the other hand, HT appearing after the first 36 h is correlated with a delayed BBB disruption. This is supported by the fact that a BBB disruption measured in the first hours of stroke does not predict the appearance of HT occurring later than 3 days (129). It is thought that late HT occurs due to increased BBB permeability and blood flow after cerebral edema reduction (130, 131). The hemorrhage appearance differs according to the stage it occurs. While early HT tends to be a dense parenchymal hematoma, late HT more frequently takes the form of petechial hemorrhages (132). Figure 3 shows a schematic representation of the main processes that lead to early and late HT.

Early BBB disruption and, therefore, early HT are driven by several molecules as expressed in the previous sections. Reperfusion-induced ROS can disrupt the neurovascular unit (66) which will lead to the entrance of neutrophils from the blood. Neutrophils have, as pointed out earlier in this review, the capacity to secrete MMP-9. Neutrophil-derived MMP-9 is therefore a pivotal mediator of early HT (133). MMP-2 is also a great determinant in early BBB disruption (134), as it has been shown in the rat model to mediate occludin and claudin-5 degradation from the cytoskeleton, causing early ischemic BBB disruption (135).

In contrast, delayed BBB disruption and HT (>36 h) are believed to be related to the activation of brain-derived proteases such as MMP-9, neuroinflammation, and factors that promote vascular remodeling such as vascular endothelial growth factor (VEGF) (66). A relationship between MMP-9 levels and the development of late HT has been established (107, 136), and some animal studies have shown that after 24 h, the major source of MMP-9 is brain cells and not neutrophils (137, 138). In parallel, vascular remodeling factors can also contribute to the development of delayed HT (66). Vascular remodeling is a key process for lesion recovery, but its initial phase requires the mobilization of progenitor endothelial cells motivated especially by VEGF. This process implies an immature BBB (139), hence the risk of developing HT. This mechanism will be further explained later in this manuscript.

### HT Motivated by Reperfusion Treatments: Thrombolysis and Endovascular Treatment

As said, reperfusion is a crucial step for a favorable outcome after stroke. But although reperfusion treatment is absolutely necessary for tissue survival, it also contributes to additional tissue damage (47, 78) that can cause HT, which will lead to a worse clinical outcome and patient recovery. Mechanisms behind rTPA-induced HT include thrombolytic and non-thrombolytic actions. It has been suggested that after rTPA therapy, HT is motivated not only by reperfusion but also due to the dysregulation in extracellular proteolysis of the NVU matrix through tPA's effects on metalloproteinase activity (66). In fact, MMP-9 release from human neutrophils is highly induced by rTPA (7), which is in line with the previously described mechanism of early HT. In addition, the contrast used in imaging and therapeutic acute phase techniques may itself promote hemorrhage. Toxicity on basal lamina is thought to be the underlying mechanism (78).

Endovascular treatment (EVT) in stroke care poses additional challenges. Firstly, mechanical clot removing implies, at least partially, direct endothelial trauma and potential disruption. Devices used in mechanical thrombectomy (MT) lead to endothelial denudation, disruption of the internal elastic lamina, and edema in the intimal and medial layers (140). Secondly, it allows for rapid and sudden reperfusion. Not surprisingly, HT is a major complication of EVT. Symptomatic intracranial hemorrhage (ICH) occurred in 4.4% of patients in HERMES meta-analysis (141), and Hao et al. meta-analysis (142), including 1,499 patients submitted to EVT who showed that 35% of them developed ICH. Several predictors of HT after EVT have been identified so far. At time to the procedure, low ASPECTS score and poor collateral status are among them (143–145). These have also been associated with edema formation (146). There is a close interaction between BBB and EVT. Hyperdensities visible on post-procedural computed tomography after EVT are common and may be secondary to contrast extravasation or ICH. Both are due to a state of increased BBB permeability or even disruption. The contrast usually clears up within 24 h, though some studies point out an association between HT and unfavorable outcome (147). Dual-energy head CT is currently the gold standard to distinguish both conditions in control CT post-EVT (147, 148). One study (123) found BBB disruption evaluated by non-contrast CT scan within 3 h of the procedure and defined as parenchymal hyperdensity on CT scan (including both blood and contrast medium), occurring in 61% of patients. Multiple thrombectomy passes have also been independently associated with a significant increase in BBB disruption as evaluated by hyperintense acute reperfusion marker (HARM) which is a hyperintensity on T2-weighted fluid-attenuated inversion recovery (FLAIR) MRI following an injection of gadolinium-based contrast agent (149). An interesting question raised by Renú et al. (150) regarding such an imagological finding is whether this is attributable to MT-induced damage to BBB at a microcirculatory level or by direct damage to the proximal vessel wall where the MT device is deployed. The same question can be raised to iodinated contrast extravasation and the associated BBB compromise mentioned above.

There is no firm evidence on the actual HT rates for each of the stroke phases, but there is a trend toward a higher rate in the acute phase, particularly when reperfusion therapies are administered and when reperfusion is achieved outside the desirable time window. In fact, study comparisons are significantly hampered by different methodologies, time-point assessments, classification, and imaging modalities used. However, it is consensual that the vast majority of symptomatic HT post-reperfusion seem to occur within 24 h, and only around 10–15% occur beyond this time, with the clear majority occurring within 36 h (151). In a study compromising 55 stroke patients, the rate of HT in the acute phase (first 60 h) was 62.5% for rtPA-treated patients and 33.3% for the non-treated group of patients. On the opposite, 37.5% of the treated patients developed HT beyond 60 h, this being at a rate of 66.67% of the non-treated patients (152). In line with this, another study of 30 AIS patients who developed HT showed that 40% of them presented with HT in the first week with a median of 24 h, and 60% developed HT beyond 1 week, with a median of 21 days. Of the patients presenting with HT in the acute phase, 25% have had received reperfusion therapy, while only 16.7% of the late HT patients did (131). In another cohort of 527 patients, 86.3% of patients with HT developed it in the first 48 h (130). Montaner et al. showed that 68.75% of patients with HT presented it in the first 48 h. Interestingly, seven of the 11 patients developing HT in the first 48 h had recanalization in the first 12 h, while none of the patients with HT beyond 48 h had early recanalization (110). In line with this work, Molina et al. showed, in a cohort of non-thrombolytic treated patients, that a spontaneous recanalization happening between 6 and 24 h directly affected the appearance of HT. In this study, 58% of patients that underwent recanalization between 6 and 12 h developed HT; this incidence was 50% for patients recanalizing at 12–24 h, while patients that had spontaneous recanalization in 6 h or less and between 24 and 48 h did not develop any HT (91). In another cohort of 407 patients with AIS not receiving thrombolytic treatment, the authors found spontaneous HT in 12.3% of the patients, 32% within the first 48 h and 40% between 4 and 7 days from the symptom onset (153).

An absolute HT rate for each stroke phase is difficult to establish considering the multitude of factors influencing its development, but it seems clear that recanalization occurring after the hyperacute phase is directly linked with the early appearance of HT, while the lack of recanalization may motivate a delayed appearance of HT. Nonetheless, patient and stroke characteristics such as stroke severity and infarct size, increasing age, baseline systolic blood pressure, hypertension, or serum glucose (66) are factors influencing the appearance of HT and its time-point.

## Blood–Barrier Permeability Evaluation in the Early Detection of HT

Due to the lack of effective treatment, the early prediction of HT has become one of the hot topics in stroke research aiming to find a better patient selection for recanalization therapies. As discussed before in this manuscript, BBB breakdown is considered to be the basic pathophysiology of HT, and BBBP is directly correlated with the appearance of subsequent HT, suggesting that the assessment of the BBB permeability should be the most promising predictor of HT (154). The most common tools to measure BBBP are imaging techniques due to their accessibility and capability to quantitatively assess permeability. Nonetheless, biomarkers evaluating BBBP may also enable the early assessment of BBB disruption and hence the early prediction of HT (154) in AIS patients. In this section, we will review the most used and promising tools for BBBP assessment and HT prediction, each of which has its own advantages and disadvantages (Table 1).

**Table 1:** Advantages and disadvantages of blood–brain barrier permeability assessment methods.

**Method**	**Advantages**	**Disadvantages**	**References**
**IMAGING**			
Dynamic contrast-enhanced mri	Quantitative measure No biological risk Not invasive Good temporal resolution	Expensive Contrast agent needed Not widely available Potential patient limitation (e.g., metal implants)	(42, 155–157)
Dynamic susceptibility contrast mri	No biological risk Not invasive Very short acquisition time	Similar to DCE-MRI	(42, 156, 158)
Computed tomography	Quantitative measure Cheap and fast Widely available Acquisition and processing simplicity High-resolution parametric images	Contrast agent needed Limited brain coverage Ionizing radiation Longer bolus duration than MRI	(42, 155–157)
Positron emission tomography	Quantitative hemodynamic data	Expensive Radioactive Highly inaccessible and time-consuming Complex procedures Poor spatial and temporal resolution	(159, 160)
**BIOMARKERS**			
mmp-9	High sensitivity for BBB damage Low cost Quantifiable	Single time-point Results are not reflected immediately Low specificity for acute ischemic stroke (AIS)	(161)
S100B	High sensitivity for BBB damage Low cost Quantifiable Stable in the bloodstream	Low specificity for AIS Not immediately reflected Single time-point	(161, 162)
Tight junction proteins	Prospective marker Low cost Quantifiable	Single time-point Results are not reflected immediately	(161)
Cerebrospinal fluid/plasma albumin ratio	Low-cost, method-independent measure: allows the same reference value for different institutions Quantifiable	Invasive Not commonly used	(161, 163)

### Imaging Tools

BBB integrity can be evaluated in a timely manner by MRI or CT imaging. Both modalities detect the extravasation of intravenously administered gadolinium or iodine-based contrasts, respectively. BBB status can be not only qualitatively (statically) but also quantitatively (dynamically) evaluated depending on the inclusion of time as a variable of interest and the attenuation or enhancement pattern following the administration of contrast agent as the nominators. Static imaging is limited to a binary evaluation of signs and patterns secondary to contrast extravasation. Such signs include parenchymal enhancement on post-contrast T1-weighted MRI, which is highly specific but not a sensitive predictor of HT (155, 164), HARM sign, and hyperdensities on CT scan. These latter findings have been mentioned earlier in this manuscript. In short, HARM corresponds to the juxtacortical cerebrospinal fluid (CSF) enhancement on FLAIR imaging and has been associated with HT in AIS patients (128, 165). This association, however, has been contested (166, 167). Hyperdensities seen in control non-contrast CT scan after MT consist of early intraparenchymal hyperdense areas as a result of increased permeability and may predict HT after MT (123).

Currently, dynamic contrast-enhanced (DCE) MRI is considered the most widely used imaging technique for BBB research (168) since it provides quantitative estimates of contrast agent leakage at moderate spatial resolution. It allows for a quantitative BBBP assessment both in healthy and diseased vessels with an adequate spatial resolution. Predicting HT has been the main clinical research application of DCE-MRI studies in AIS. Its time-consuming protocols for image acquisition makes DCE-MRI still not part of the routine clinical practice (169, 170). In this sense, dynamic susceptibility contrast (DSC) MRI is one of the most appealing alternatives. DSC is frequently used in stroke imaging (170) and, like DCE, relies on the tracking of a paramagnetic intravenous contrast agent. It is based on the susceptibility changes after injecting the contrast agent and depends only on its first pass, therefore reducing the image acquisition time (158, 169). DSC-MRI can be referred to as perfusion-weighted imaging (PWI), providing information about the blood flow to the brain (169) and which can be used as a surrogate for BBBP able to predict HT (42).

Perfusion CT can also generate BBBP maps that can help in the identification of patients at risk of HT (65, 171, 172), but its relevance compared to other predictors has been contested (173).

Not so relevant in an acute stroke context due to its accessibility and time consumption, positron emission tomography scan allows the determination of BBBP by the uptake of tracers radiolabeled with short-lived positron-emitting isotopes with a lower spatial resolution compared with MRI but with higher sensitivity (174, 175).

So far, we are unable to withhold patients from reperfusion therapies based on BBB status evaluated from these imaging techniques, but imaging of the BBB in the early hours of stroke is a promising strategy to improve patient safety. Also promising is the usage of these imaging modalities in the selection of patients that may benefit from an extended time window of intravenous thrombolysis (176) and in the early detection of patients that may develop severe stroke complications such as HT, which would be key in the specific guided treatment of AIS patients avoiding, therefore, poorer prognosis and adverse clinical developments (154).

A recent meta-analysis on the prognostic performance of MRI on HT prediction in AIS patients showed that MRI has a specificity of 79% and a sensitivity of 82%. These values increased when perfusion imaging was studied alone, with 92% specificity and 80% sensitivity in terms of HT prediction (126).

DSC-PWI permeability values have been used to try to establish a relation between BBBP and subsequent HT. Pretreatment MRI PWI with DSC imaging from a cohort of patients from DEFUSE 2 trial was studied. The investigators found a relationship between the degree of BBB disruption and the severity of ICH. A pre-specified BBBP disruption threshold of 21% resulted in a sensitivity of 37.5% and a specificity of 80% for the prediction of parenchymal hematoma, with a positive predictive value of 0.375 (177). Similarly, another study with PWI imaging showed pretreatment permeability derangements to have 29% sensitivity and 98% specificity in predicting HT (178).

Regarding CT, a meta-analysis on its predictive value revealed that a high BBBP derived from perfusion CT analysis is associated with the appearance of HT, with a pooled sensitivity of 84%, a pooled specificity of 74%, an estimated positive predictive value of 46%, and an estimated negative predictive value of 77% (127). In line with this, another meta-analysis on the predictive value of perfusion CT showed a pooled sensitivity of 85.9% and a pooled specificity of 73.9% (95% CI: 45–92%) and an accuracy with a negative predictive value of 92.9% (179).

### Biomarkers

So far, many biomarkers have been reported to indicate BBB damage, but none of them meets all the characteristics for an ideal biomarker (high specificity, sensitivity, and reliability, easy and fast assessment, and minimal invasiveness) (161). Here we present a brief review of the most promising biomarkers in terms of BBBP assessment and HT prediction.

#### MMP-9

MMP-9 plasma concentration is a strong marker of BBB disruption and permeability in stroke. High plasma MMP-9 concentration levels have also been proven to be an independent predictor of HT. MMP-9 plasma levels of ≥140 ng/ml measured in AIS patients showed a sensitivity of 87% and a specificity of 90% for HT prediction, with positive and negative predictive values of 61 and 97%, respectively (180). Recently, it has been shown that the levels of MMP-9 over 181.7 ng/ml have 82.9% sensitivity and 81.3% specificity for the prediction of spontaneous HT in non-treated patients (181) and that values over 775 ng/ml measured at 6 h are independently associated with HT [OR 2.91 (1.14–7.42); *p* = 0.03] in patients treated with thrombectomy (182).

#### S100β

S100β is a low-molecular-weight glial protein (45) that does not circulate in the blood of healthy individuals, but it can be released and detected in the peripheral blood after BBB injury; hence, its concentration is related to the extent of BBB opening (161, 183). Its presence after AIS has been directly correlated with subsequent HT. A study performed in patients treated with thrombolytic therapy found that a cutoff value of 0.23 μg/L of S100β provided a sensitivity of 46% and a specificity of 82% in the independent prediction of HT after AIS (184). This is in line with another study presenting a cutoff value of >11.89 pg/ml of S100β as a predictor of HT in non-treated AIS patients, with 92.9% sensitivity and 48.1% specificity (185).

#### TJs Proteins

BBB components, particularly TJ proteins such as claudin 5 and occludins, can also serve as BBBP biomarkers due to their release into the blood circulation after stroke (161). Analyzing serum levels of BBB components may be an effective way to screen for subsequent HT (154). A great amount of the BBB's selective permeability has been attributed to claudins, with claudin-5 being the most abundant type in the BBB; therefore, the appearance of this protein in the serum of AIS patients is thought to be indicative of increased BBBP (186). In addition, significantly higher levels of claudin-5 have been found in patients with HT in comparison with those without HT, with a predictive cutoff value for HT of >1.601 ng/ml, sensitivity of 64.3%, and specificity of 53.9%. These predictive values are similar to occludins. With a cutoff value of >0.029 ng/ml, occludins yield 58.6% and 67.5% of sensitivity and specificity, respectively (185).

#### Cerebrospinal Fluid/Plasma Albumin Ratio

The CSF albumin concentration is minimal in non-pathologic conditions. After BBB disruption, albumin can enter CSF from the blood; consequently, the CSF/serum albumin ratio can be used as a reliable marker of BBBP (6, 161). It has been shown that AIS patients have higher values of this ratio than healthy people (163, 187, 188). The CSF/plasma albumin ratio has also been related to the severity of the injury, stroke evolution, and long-term outcome (163). In addition, this ratio has been shown to be associated with the appearance of HT in AIS patients (188). Despite that, the acquisition of this ratio requires obtaining both blood and CSF albumin, limiting the use of this BBBP biomarker (161).

The predictive value of protein biomarkers for HT varies widely; hence, it has been proposed that a panel of biomarkers could have greater discriminative power than any single biomarker alone (188).

## The Blood–Brain Barrier in the Subacute Stage of Stroke

The subacute stage takes place around 1 week post-stroke, being crucial in brain repair and patient recovery.

Brain recovery is dependent on neuroinflammation. As we have described in the acute phase, neuroinflammation is mediated in a great part by pro-inflammatory microglia, leading to pathologic inflammation. Recently, it has been suggested that this harmful active microglia of the acute stage may have beneficial effects when it appears in delayed stages (100). The neuroinflammatory reaction will become a recovery pathway by changing microglia to its anti-inflammatory phenotype. Anti-inflammatory microglia contributes to stroke recovery by expressing anti-inflammatory cytokines, such as IL-10, IL-4, and some neurotrophic factors which prevent inflammation (100) and play important roles in tissue repair and wound healing (27).

All of these recovery processes lead to a stabilization of the permeability of the BBB, although several animal studies have shown increased permeability up to 1 (62) and 3 weeks (61). Along with this, some human studies have also shown increased BBB up to 1 week (63, 68) and further (189), suggesting that BBB remains open and permeable through this subacute stage. Nonetheless, the dynamics and the permeability range of this phase are extremely diverse and dependent on the stroke characteristics (47).

The regenerative response after stroke includes angiogenesis and the modification of the vascular tree (54), the former being one of the major events contributing to an increase in BBB permeability in the subacute stage. An association of subacute BBB permeability with the migratory and angiogenic capacities of endothelial progenitor cells (EPCs) at day 7 after stroke has been shown in humans (68).

### The Importance of Angiogenesis in Stroke Recovery and BBB Permeability

Angiogenesis is a multi-step process that refers to the sprouting of new blood vessels from the already existing vasculature (190). It is a natural physiologic mechanism that restores blood flow and, hence, oxygen supply, and normal metabolism in the ischemic tissue (191) being fundamental for ischemic brain repair (192). Promoting angiogenesis is one of the most important strategies for functional recovery after stroke (193). It is closely associated with reduced cerebral infarction and improved neurological recovery (194) as shown in animal models of stroke (195) and AIS patients (136). In fact, higher angiogenesis has been associated with a longer survival of stroke patients (196) and BBB stability (136). However, its benefit is time dependent, as the premature promotion of angiogenesis after stroke (such as VEGF administration) can lead to enhanced vascular permeability and increased HT risk (197).

Angiogenesis is a complex process that involves several consecutive steps (198) from endothelial cell proliferation and migration to tube formation, branching, and anastomosis (192, 198). All these processes are modulated by the inflammatory microenvironment formed in previous stages (105). Therefore, the induction of angiogenesis after an ischemic injury is mediated by the same stimuli that cause the pathologic BBB disruption (198). BECs are the primary effectors of the angiogenic response after ischemic injury, followed by the pericytes and smooth muscle cells (139).

In the first moment, angiogenesis requires the mobilization of EPCs to the ischemic area (105). This needs vasodilatation and permeability increase of the existent vessels and happens subsequently after hypoxia, in response to the production of chemokines and factors such as VEGF and ANG-2 by hypoxic cells (199). BECs enlarge and produce proteases (collagenase and matrix metalloproteinase) that are capable of local degradation of the basement membrane (139). Pericytes detach from the vessel wall and liberate themselves from the basement membrane, thanks to proteolytic degradation (199). This implies the initial rupture of the BBB for cellular migration. Once this mobilization is completed, BECs return to a quiescent-like state that is associated with the formation of cell–cell junctions and vessel maturation (200). In animal models of stroke, endothelial proliferation has been reported as early as 12–24 h (139), while active angiogenesis, with the consecutive maturation and stabilization of blood vessels, has been proven to occur around 3–4 days after injury in AIS patients (196).

VEGF, from various cellular sources, binds to its receptors on nearby vascular endothelial cells to directly initiate an angiogenic response. This binding activates a series of downstream signals and tyrosine kinases that promote angiogenesis (194). Angiopoietin (Ang)-1 and Ang-2 and their receptors, Tie-1 and Tie-2, are also deeply involved in neoangiogenesis. In short, Ang-2 promotes angiogenesis, whereas Ang-1 inhibits it (194). The close regulation between VEGF and angiopoietins plays a pivotal role in neoangiogenesis. Zhang et al. demonstrated in a rat model of stroke that the early upregulation of VEGF receptors along with a downregulation on Ang-1 is linked to an increase in BBB leakage. On the contrary, the upregulation of VEGF receptors and Ang/Tie 2 in later stages was correlated with the increase in the number of capillaries and enlarged vessels in the penumbra (201). Interestingly, it has been shown that miRNA miR-210 is involved in this angiogenic regulation in response to ischemic injury since its upregulation is able to improve angiogenesis for brain tissue repair (202).

Recently, it has been suggested that MMP-9 may also play an important role in angiogenesis. Despite its huge involvement in BBB disruption and development of HT, MMP-9 seems to be beneficial in vascular remodeling (194). Zhao et al. demonstrated in a rat model of stroke that a second phase of increased MMP-9 at 7–14 days post-stroke was correlated with angiogenesis since the inhibition of MMP-9 resulted in, among other things, malformation of blood vessels (109).

## Blood–Brain Barrier Permeability in the Chronic Phase of Stroke

In a chronic post-stroke setting, >6 weeks after the event, the permeability of BBB is significantly less compared to those of the initial phases. There is a sealing of the barrier with an overexpression of TJ proteins and a *de novo* organization of junction proteins (3). As many of the factors responsible for the hyperpermeability tend to decrease in this stage, there is also an increase in factors that contribute to the restoration of BBB permeability, such as Ang-1, which keeps endothelial cells in a quiescent state and promotes cell–cell and cell–extracellular matrix interactions, sphingosine-1- phosphate, and activated protein C, both of which stabilize junctions and the cytoskeleton (3, 203). In the long-term, angiogenesis occurring predominantly in the subacute stage allows blood flow restoration to previously ischemic areas and reduces BBB permeability by contributing to the reduction of brain-derived factors that increase BBB permeability (204). In the late phase of cerebral ischemic injury, recovery is dependent on the restoration of the NVU complex. Herein BBB development is intrinsically connected with neurogenesis. There are two endogenous neural progenitor cell hubs in the adult brain: in the subventricular zone (SVZ) of the lateral ventricle walls and in the sub-granular zones of the hippocampal dentate gyrus and circumventricular organs (205–207). Neurogenesis is linked with angiogenesis. Cells in SVZ have increased expression of VEGF and its receptor Flk. In fact, VEGF is both an angiogenic and a neurotrophic factor that contributes to the migration of neuronal progenitor cells (208, 209). Being the most abundant cell type within the brain parenchyma, astrocytes have a predominant role in the regulation of neurogenesis. They are the source of two major astrocytic intermediate filament proteins, glial fibrillary acidic protein and vimentin, whose absence would severely impact stroke recovery (210). Several neurotrophic factors are also secreted by astrocytes, including brain-derived neurotrophic factor, glia-derived neurotrophic factor, nerve growth factor, basic fibroblast growth factor, VEGF, ciliary neurotrophic factor, and erythropoietin (6). These astrocyte-derived factors not only protect neurons in the acute phase but also play a crucial role in promoting neuron survival, axonal sprouting, neurovascular unit remodeling, and functional recovery in the chronic phase by contributing to neural repair and neuroplasticity (3, 6). Astrocytes have shown *in vitro* to stimulate the migration and proliferation of adult neural stem cells through the secretion of the neuroblast attracting chemokine stromal cell-derived factor-1 (211). Synaptogenesis is controlled and enabled by astrocytes *via* thrombospondins 1 and 2, which are secreted glycoproteins that are activated after AIS. Its absence, in animal models, leads to defects in synaptogenesis and axonal sprouting post-stroke (212). Other cell types are deemed important. Progenitor cells are integrated directly in the damaged barrier, particularly in the endothelium and the surrounding tissue, and release mediators that contribute to barrier repair after stroke (6). Circulating EPC has a relevant role in the subacute phase as mentioned before. Mesenchymal stem cells (MSCs) are another category of progenitor cells and one of the leading restorative cell therapy candidates. VEGF, Ang-1, bFGF-2, placental growth factor, and insulin-like growth factor are among the variety of angiogenic factors expressed by MSC (6). Most studies have focused on MSC potential in the early stages of stroke (213), but a recent phase I/II clinical trial has evaluated the administration of intravenous allogenic MSC in chronic stroke, suggesting behavioral gains and laying the ground for a randomized controlled trial (214). Juxtavascular microglia, which are brain tissue-resident macrophages, enhance brain repair by removing toxic debris, reducing neuroinflammation and releasing trophic factors (215). This is true for the tissue-restorative microglia (M2) phenotype as opposed to the M1 phenotype. Microglial activation has been shown to occur in every phase of ischemic stroke. In the chronic phase, activated microglia are located in the peri-infarct region and distal areas, but generally the greatest amount of microglia peaks in the early stages (216). Beneficial and detrimental effects of microglia have been observed depending on the morphology and subtype of activated microglia (216). Chronic cerebral hypoperfusion induces microglial activation, polarized toward M1 (pro-inflammatory) phenotype, and the drug fingolimod has been shown, in an animal model, to attenuate such microglia-mediated neuroinflammation and to promote oligodendrocytogenesis by shifting microglia toward M2 subtype (217). Clopidogrel and other P2R2Y12-targeted antiplatelets which are frequently administered in a chronic stroke setting as means of secondary prevention affect G-protein coupled purinergic receptor P2Y. This receptor mediates the microglia-induced reversal of BBB opening after an injury (3, 218).

Even though several repair mechanisms exert its efficacy, in the long run, some degree of BBB dysfunction will persist with long-lasting leakage after stroke. Insufficient or disorganized TJ complex formation, an expression of claudin-1 with decreased levels of claudin-5 trans interaction, and claudin-5/ZO-1 interaction in leaky vessels (219) are among the proposed mechanisms behind an incomplete recovery of BBB permeability. In fact, the risk of intracranial hemorrhage after an ischemic stroke is the greatest in the first 30 days and decreases thereafter, but it remains higher than in the general population (220).

The incomplete closure of BBB, on the other hand, may benefit neurogenesis due to the link between these two processes mentioned before, and it can be seen as a window of opportunity for therapeutic delivery, as endogenous neural recovery after an ischemic stroke is often insufficient. This is the case for cell therapy in stroke recovery, more specifically a BBB permeation-mediated stem cell therapy, as the entrance of transplanted cells into the brain from the periphery is facilitated by a permeable BBB. Transplantation of allogeneic neural, mesenchymal, and endothelial stem cells has itself shown promising results in stroke models in stabilizing BBB and promoting BBB integrity and in vascular regeneration after stroke (8, 221).

### Cerebral Small-Vessel Disease

BBB dysfunction is a hallmark of cerebral small-vessel disease (cSVD), a condition associated with cognitive impairment, lacunar strokes, microinfarcts, microbleeds, and widespread white matter injury (222–226). Patients with higher white matter hyperintensities have significantly higher BBB leakage as evaluated by DCE-MRI (227). Doubts arise on the directionality of the association between BBB and cSVD (cause *vs*. consequence) and the exact location of the dysfunction (capillaries? arterioles?), but its association is undisputable (226, 228). On the basis for such association might be altered cerebral hemodynamics with loss of cerebral autoregulation, higher arterial stiffness, and increased speed and pulsatility of flow in small vessels (arterioles and capillaries). The resultant sheer stress damages the endothelium and BBB (229). Endothelial dysfunction seems to play a crucial role in the pathogenesis of cSVD and BBB dysfunction. Overexpression of inflammation markers (*e.g*., intracellular adhesion molecule) (230), C-reactive protein (231), coagulation markers (232), and hyperhomocysteinemia (233) support that hypothesis. In the upstream, chronic hypertension and diabetes (both common in cSVD) have been shown to impair cerebral blood flow and oxygenation and promote an increase in BBB permeability. Hypoxia contributes to the death of oligodendrocytes and consequent gliosis, as hypoxia-inducible factor-1 has already been identified in pathological studies of affected white matter (234). Increased MMP has also been demonstrated in the white matter of patients with vascular dementia (235). Clinically, BBB derangements in this spectrum of conditions lead to a higher risk of remote cerebral hemorrhage and HT of the infarcted area after thrombolysis in patients with cSVD (178, 236–238). Leukoaraiosis itself is an imaging finding whose presence increases the risk of HT (178).

## Discussion

BBB disruption after AIS occurs predominantly in the hyperacute and acute stages where major complications and clinical deterioration may take place. Reperfusion therapy, the only approved stroke treatment, may potentiate BBB permeability and promote HT. In this review, we have tried to give thoughtful insight into the dynamic changes of BBB permeability across the timespan of an ischemic stroke, a topic in which there is still limited knowledge but a handful of therapeutic opportunities. Table 2 summarizes the main processes driving BBB dynamics and HT after stroke. We performed a comprehensive review from bench to the bedside, linking pathophysiological processes to their therapeutic counterparts and showing how hyperacute, acute, subacute, and chronic stroke care interacts with BBB status and how clinical practice may change in the future in order to prevent HT.

**Table 2:** Main events driving blood–brain barrier (BBB) dynamics and hemorrhagic transformation after stroke.

**Stage**	**Event**	**BBB status**	**Response**
Hyperacute	Na^+^ accumulation	Intact	Cytotoxic edema
Hyperacute	Ca^+^ accumulation	Initial disruption	Glutamate excitotoxicity and mitochondria dysfunction
Hyperacute	MMPs action	Disrupted	Vasogenic edema
Hyperacute	Reperfusion treatments	Disrupted	Blood flow restoration
Hyperacute/acute	Mechanical BBB damage due to reperfusion treatments	Disrupted	Hemorrhagic transformation
Hyperacute/acute	rTPA activation of proteases	Disrupted	Hemorrhagic transformation
Acute	Neuroinflammatory response	Disrupted	Permeability increase and immune cell infiltration
Acute	MMP-9 degradation	Disrupted	Hemorrhagic transformation
Acute	Start of vascular remodeling	Disrupted	Hemorrhagic transformation
Subacute	Anti-inflammatory response	Start of the recovery	Stabilization of permeability
Subacute	Angiogenesis	Start of the recovery	Physiologic BBB increase and cerebral blood flow restoration
Chronic	Overexpression of tight junction proteins	Recovered	Sealing of the BBB
Chronic	Migration of neuronal progenitor cells	Recovered	Neural repair and neuroplasticity

Stroke care and the prognosis are highly dependent on intravenous thrombolysis or mechanical thrombectomy. These treatments are available to only a fraction of AIS patients due to their strict time and tissue window eligibility criteria. Knowledge of BBB status through current and under-development permeability imaging techniques could be an interesting strategy in AIS patients to extend such criteria safely, avoid hazardous recanalization, and reduce HT or to test other strategies such as tighter BP control in patients with BBB imaging-proven severe and extensive damage. Early prediction of HT could also be enabled by the evaluation of biomarkers related to the disruption of BBB.

If knowing BBB status could potentially help us guide acute stroke care decisions, understanding the exact mechanisms behind the BBB dysregulation and the pathophysiology leading to HT is key to come up with a direct therapeutic strategy to modulate BBB.

Avoiding excessive opening in the early stages of stroke is one of the major goals in experimental stroke treatment. Protection of the BBB in the early phases of a stroke may be useful in extending the recanalization therapy time window; hence, up-regulating of existing protective mechanisms is a possible way to achieve this. For example, a preclinical trial studying CD151, a member of the transmembrane four superfamilies that plays a key role in maintaining vascular stability, showed that its upregulation in the early phases of stroke is able to preserve BBB integrity (239), turning it into a possible therapeutic target.

Other promising targets in BBB protection include the reduction of ROS and the inhibition of MMPs in the acute phases (240). As said, the increased ROS that is produced by ischemia–reperfusion can disrupt the NVU and thus predispose the BBB to HT (240); thus, targeting ROS with free radical spin-trap agents seems a promising field in stroke treatment. The spin-trap agent NXY-059 has shown to reduce stroke impairment (241) and to confer protection toward tPA-induced hemorrhage (242) in animal models of stroke. Nonetheless, it turned out ineffective in treating clinical AIS in the first hours of stroke (243, 244). Another spin-trap agent that has recently shown clinical efficacy is edarvone. One retrospective observational study conducted in Japan has suggested that the early administration of this compound was associated with better functional outcomes and reduce HT after AIS (245), although there is data suggesting that the use of this compound may be related to increased HT (246); thus, more study is needed on this matter. Preserving mitochondrial function after stroke is another approach to inhibit ROS production and reduce ischemia/reperfusion damage. A therapeutic peptide called bendavia has recently appeared in animal models as a potential neuroprotective agent acting in the mitochondrial pathway (247). Another promising strategy in this field is the use of stanniocalcin-1 (STC-1). STC-1 is a glycoprotein-secreted hormone with antioxidant effects that may reduce the formation of ROS (248) and exert neuroprotective effects against cerebral ischemic injury (249). Recently, it has been shown in an animal model of stroke that STC-1 can exert its antioxidant activity by inhibiting brain edema and BBB permeability and consequently improving the neurological alterations following cerebral ischemia (250). It may represent a potential new strategy to target stroke pathophysiology.

Similarly, early administration of MMP inhibitors has shown to reduce BBB permeability and the rate of HT (66). Since MMPs are the primary mediators of BBB disruption and hence HT, its inhibition at the early stages of stroke is of great relevance. Nonetheless, delayed MMP inhibition was shown to increase brain injury and worsen outcomes (109). Most efforts are being put into MMP-9 inhibition as it is the key mediator in ECM degradation, BBB disruption, and HT development. Minocycline, a broad-spectrum antibiotic with anti-inflammatory and antiapoptotic effects (251), is a potential neuroprotective therapeutic compound that may limit BBB dysfunction, preventing TJP disruption through the reduction of microglia's pro-inflammatory cytokines (104) and the inhibition of MMP-9 action. It has been shown to reduce HT in rat stroke models (252). The translation to the bedside of this therapeutic compound has shown to be safe in humans and effective in lowering MMP-9 levels in treated patients (253, 254), with some neuroprotective effects (255), but it does not yield firm evidence on HT prevention.

Developing novel stroke therapies is essential as most patients are ineligible for reperfusion therapy. The brain represents a therapeutic “sanctuary,” restricting the vast majority of therapeutic compounds from accessing it (256). Therefore, the selective regional BBB permeability after stroke may be used for therapeutic delivery. Nowadays, crossing the BBB remains one of the most challenging tasks for brain disease treatments and is a hot topic in the study of neurological disorders. Current strategies focus not only on targeting the BBB endothelium but also on the effective transport of specific compounds across it and the subsequent targeted drug release at appropriate targets in the brain (32).

Strategies under study to cross the BBB include viral vectors (257) as gene therapy delivery, enhancement of brain permeability (258), delivery through the pathologic permeable BBB, intranasal drug administration to bypass the BBB (259), nanoparticles (NPs) to actively target the brain, or delivery through active BBB transporters (39). These last two strategies are tightly related since transporter-mediated endocytosis of NPs through the BBB is one of the most promising pathways for drug delivery through the BBB (39). In fact, NP-mediated drug delivery is emerging as an effective and non-invasive system to treat brain diseases. Nanotechnology-based BBB crossing has been successfully demonstrated in preclinical animal studies of different neurological disorders such as Alzheimer's disease (260), brain cancer (261), and even stroke (262).

Along with NPs, using the BBB permeability as a gate to the brain is a promising tool for the delivery of targeted therapeutics in diseases related to damaged BBB, such as stroke. To reach that, further research is required to establish treatments directed to the maintenance and modulation of the BBB integrity through the inhibition/enhancement of the underlying biochemical processes.

Another important target in stroke therapy is neoangiogenesis occurring in the subacute stage. New vessels forming due to angiogenesis make the BBB leakier, and although such remodeling is necessary for brain recovery as already commented, as the vessel becomes leakier, they can be prone to HT due to this vascular unsteadiness (240). In fact, a study performed in an animal model of stroke showed that the treatment with anti-VEGF reduced the incidence of HT along with attenuated degradation of BBB components and MMP-9 activation even in the presence of tPA treatment (263), suggesting that the modulation of neoangiogenic factors could be an interesting strategy to avoid or lessen HT.

Although still in pre-clinical research, a very promising approach for stroke treatment is gene therapy through the use of miRNAs due to their ability to regulate large sets of genes related to different pathways of the ischemic stroke cascade (55). Several recent studies have shown the possibilities in this field. Bernstein et al. demonstrated that, in an animal model of stroke, the early administration of the let-7g^*^ miRNA was capable of preserving neural tissue, diminishing BBBP, and ameliorating the neuroinflammatory immune response (264). Another recent study showed that the overexpression of the miRNAs miR-126-3p and−5p was able to reduce the expression of proinflammatory cytokines and adhesion molecules and hence attenuate the BBB disruption in the acute stage of AIS (265). Sun et al. demonstrated that the selective deletion of the miRNAs miR-15a/16-1 decreased BBBP, macrophage infiltration, and brain water content, suggesting that the pharmacological inhibition of this miRNA cluster could be a promising target in genic stroke therapy (266).

Modulation of the BBB and/or targeted delivery strategies, either taking advantage of this modulation or using the physiological transport pathways to deliver therapeutic compounds into specific parts of the brain, are under extensive study in a pre-clinical setting. Unfortunately, the majority of promising therapies in animals have failed to successfully translate into clinical therapies for HT (66), and we are still lacking applicable translational therapeutic options to reach the CNS.

What may be more within reach is the evaluation of BBB permeability through perfusion studies in AIS patients as a way of avoiding HT as well as including those that would otherwise be ineligible to reperfusion therapies, leaving aside strict and rigid criteria such as time and moving toward a more personalized medicine.

## Author Contributions

JS-F was responsible for the conception of the general idea of this work. SB-C and JS contributed to the refinement and modulation of the manuscript structure, conducted the literature review, and wrote the manuscript. JS-F critically revised the work for intellectual content. The rest of the co-authors critically revised and corrected the final version of the manuscript.

## Conflict of Interest

The authors declare that the research was conducted in the absence of any commercial or financial relationships that could be construed as a potential conflict of interest.

## Abbreviations

AIS: acute ischemic stroke
AJs: adherens junctions
AMT: adsorptive-mediated transcytosis
Ang: angiopoietin
ADC: apparent diffusion coefficient
ABC: ATP-dependent binding cassette
BM: basement membrane
BBB: blood–brain barrier
BECs: blood–brain barrier endothelial cells
BBBP: blood–brain barrier permeability
CMT: carrier-mediated transport
CNS: central nervous system
CBF: cerebral blood flow
cSVD: cerebral small-vessel disease
SDF-1: chemokine stromal cell-derived factor-1
Cx: connexin
DW-MRI: diffusion-weighted magnetic resonance imaging
DCE: dynamic contrast-enhanced
DSC: dynamic susceptibility contrast
EPCs: endothelial progenitor cells
EMCs: extracellular matrix components
GJs: gap junctions
GFAP: glial fibrillary acidic protein
HT: hemorrhagic transformation
HARM sign: hyperintense acute reperfusion marker
ICH: intracranial hemorrhage
JAMS: junctional adhesion molecules
LAT: large neutral amino acid transporters
MIP-1α or CCL3: macrophage inflammatory protein1-α
MMP: matrix metalloproteases
MSCs: mesenchymal stem cells
MT: mechanical thrombectomy
MCA: middle cerebral artery
MCP-1 or CCL2: monocyte chemotactic protein-1
NVU: neurovascular unit
OAPs: orthogonal arrays of particles
PWI: perfusion-weighted imaging
ROS: reactive oxygen species
RMT: receptor-mediated transcytosis
rTPA: recombinant tissue plasminogen activator
CCL5 or RANTES: regulated upon activation, normal T-cell expressed and secreted
SVZ: subventricular zone
TJs: tight junctions
VEGF: vascular endothelial growth factor
WMH: white matter hyperintensities.
